# Unusual Cause of Distress in an Intellectually Disabled Adolescent: A Reminder to Suspect Foreign Body Ingestion

**DOI:** 10.1002/ccr3.72254

**Published:** 2026-03-11

**Authors:** Riya Thapa, Utsav Sitaula, Raju Basnet, Ajay Kumar Yadav, Gyanendra Bahadur Malla

**Affiliations:** ^1^ B.P. Koirala Institute of Health Sciences Dharan Nepal; ^2^ Department of General Physician and Emergency Medicine B.P. Koirala Institute of Health Sciences Dharan Nepal

**Keywords:** atypical presentation, dysphagia, emergency medicine, esophageal impaction, foreign body ingestion, intellectual disability, neurobehavioral agitation, nonverbal patient

## Abstract

Foreign body ingestion is a common yet often underdiagnosed condition in intellectually disabled, nonverbal patients due to atypical presentations. Early recognition is essential to prevent serious complications. A 17‐year‐old nonverbal boy with intellectual disability presented with abnormal posturing and agitation, initially suspected to be a seizure episode. Radiological imaging revealed a large, round, radiopaque object in the esophagus, later identified as a whole lemon. The object was successfully displaced distally during endoscopic intervention. In patients with limited communication abilities, foreign body ingestion can mimic neurological or behavioral disorders. Maintaining a high index of suspicion, along with prompt radiological evaluation and timely endoscopic intervention, is critical to prevent airway compromise and other complications. Clinicians should consider foreign body ingestion in intellectually disabled or nonverbal patients presenting with unexplained neurobehavioral symptoms. Early diagnosis and intervention are crucial for favorable outcomes.

## Key Clinical Message

In intellectually disabled or nonverbal patients, atypical manifestations such as neurobehavioral agitation, abnormal posturing, drooling, dysphagia, or unexplained oropharyngeal discomfort should raise suspicion for foreign body ingestion. Early radiological evaluation and timely endoscopic intervention are essential for accurate diagnosis, prompt treatment, and prevention of potentially serious complications.

- Foreign body ingestion may present atypically in intellectually disabled andor non‐verbal patients, often mimicking behavioral or neurological disorders.
- Symptoms such as abnormal body movements, drooling of saliva, agitation, difficulty in swallowing, and throat discomfort in intellectually disabled individuals presenting to the ER should raise suspicion for esophageal impaction.
- Detailed history may be unavailable in such patients, necessitating a high index of clinical suspicion.
- Early radiological imaging is crucial for timely diagnosis, especially in the absence of classical symptoms.
- Prompt endoscopic intervention within 24 hoursh can prevent complications and improve clinical outcomes.

## 1 Introduction

Foreign body ingestion is defined as the unintentional or intentional swallowing of any object not meant for consumption. It is a relatively common clinical issue encountered in emergency departments in Nepal, particularly among pediatric patients, older adults, psychiatric patients, and individuals with intellectual disabilities. The estimated annual incidence rate is 13.0 per 100,000 population [1].

Most ingested foreign bodies pass through the gastrointestinal (GI) tract without causing symptoms and result in only minor injury. However, surgical intervention is required in approximately 16.42% of cases due to obstruction [2]. In adolescents with cognitive impairment, the risk is heightened due to behavioral tendencies and a limited ability to express symptoms effectively, often leading to delayed diagnosis and management. This delay increases the risk of complications such as gastrointestinal obstruction and perforation.

Plain radiography (X‐ray) is typically the first‐line diagnostic tool. In more complex cases, endoscopy is indicated. The site of impaction depends on the shape and size of the foreign object, as well as the anatomical features of the GI tract. Impaction most commonly occurs at physiological narrowings of the esophagus.

Potential complications include mucosal injury, esophageal perforation, mediastinitis, stricture formation, airway compromise, aspiration pneumonia, pressure necrosis, bleeding, and abscess formation.

Clinicians must maintain a high index of suspicion, especially when managing cases of unexplained distress or gastrointestinal symptoms in intellectually disabled individuals. Here, we report a case of an intellectually disabled adolescent male with a whole lemon lodged in the esophagus.

### 1.1 Rationale

Foreign body ingestion is a well‐recognized emergency; however, its diagnosis becomes significantly more challenging in patients with intellectual disabilities and limited ability to communicate effectively. These individuals often present with nonspecific or atypical symptoms that may mimic neurological or behavioral conditions, leading to delays in appropriate management.

In this case, the patient's initial presentation resembled a seizure episode. However, what shifted clinical suspicion was not only his behavioral history of mouthing tendencies but also the presence of soft tissue trauma on the oral mucosa. Scratch marks were noted while the patient persistently attempted to insert his fingers into his mouth, presumably trying to extract the impacted object. This unusual behavior and injury pattern raised suspicion of possible foreign body impaction.

Keen observation by an experienced clinician led to timely imaging and endoscopic intervention. This case was chosen for reporting to emphasize the critical importance of clinical intuition, multidisciplinary evaluation, and early diagnosis in intellectually disabled patients with communication barriers. It also highlights the need for increased awareness among caregivers and healthcare professionals, especially in low‐resource settings like eastern Nepal, where such cases may be under‐recognized or misdiagnosed.

## 2 Patient Information

A 17‐year‐old male with a known history of intellectual disability was presented to the Emergency Department, accompanied by his mother with a 2‐h history of abnormal body movements, drooling of saliva, agitation, difficulty in swallowing, and throat discomfort for the past few hours. There was no antecedent history of trauma, fever, or similar previous episodes requiring hospital admission. No signs of respiratory distress, cyanosis, or stridor were observed at presentation. His mother reported that he had been banging his head against the wall a few times as these symptoms developed.

### 2.1 Clinical Findings

On initial examination, soft tissue scratch marks and superficial abrasions were noted around the patient's mouth and oral mucosa which were presumed to be self‐inflicted. His vital signs were as follows: Pulse rate 100 bpm, respiratory rate 28 breaths per min, SpO_2_ 90% on room air, and random blood sugar (RBS) 113 mg/dL. He was triaged as Australasian Triage Scale (ATS) category 2 and placed in the resuscitation bay. The patient was conscious but irritable, showing intermittent tonic posturing of the upper limbs, drooling, and inability to speak or swallow.

## 3 Methods

### 3.1 Differential Diagnosis

The initial working diagnosis was a seizure disorder due to the patient's neurobehavioral symptoms, including tonic posturing. However, a senior clinician observed that the pattern of symptoms was atypical for seizure, and also due to the presence of self‐inflicted oral injuries, he recommended evaluation for a possible foreign body. Further history revealed the patient had a known tendency towards mouthing behavior, supporting the suspicion. Additionally, the mother's observation of head banging due to distress strengthened the clinical impression of possible esophageal impaction.

## 4 Diagnostic Assessment

**FIGURE 1:**
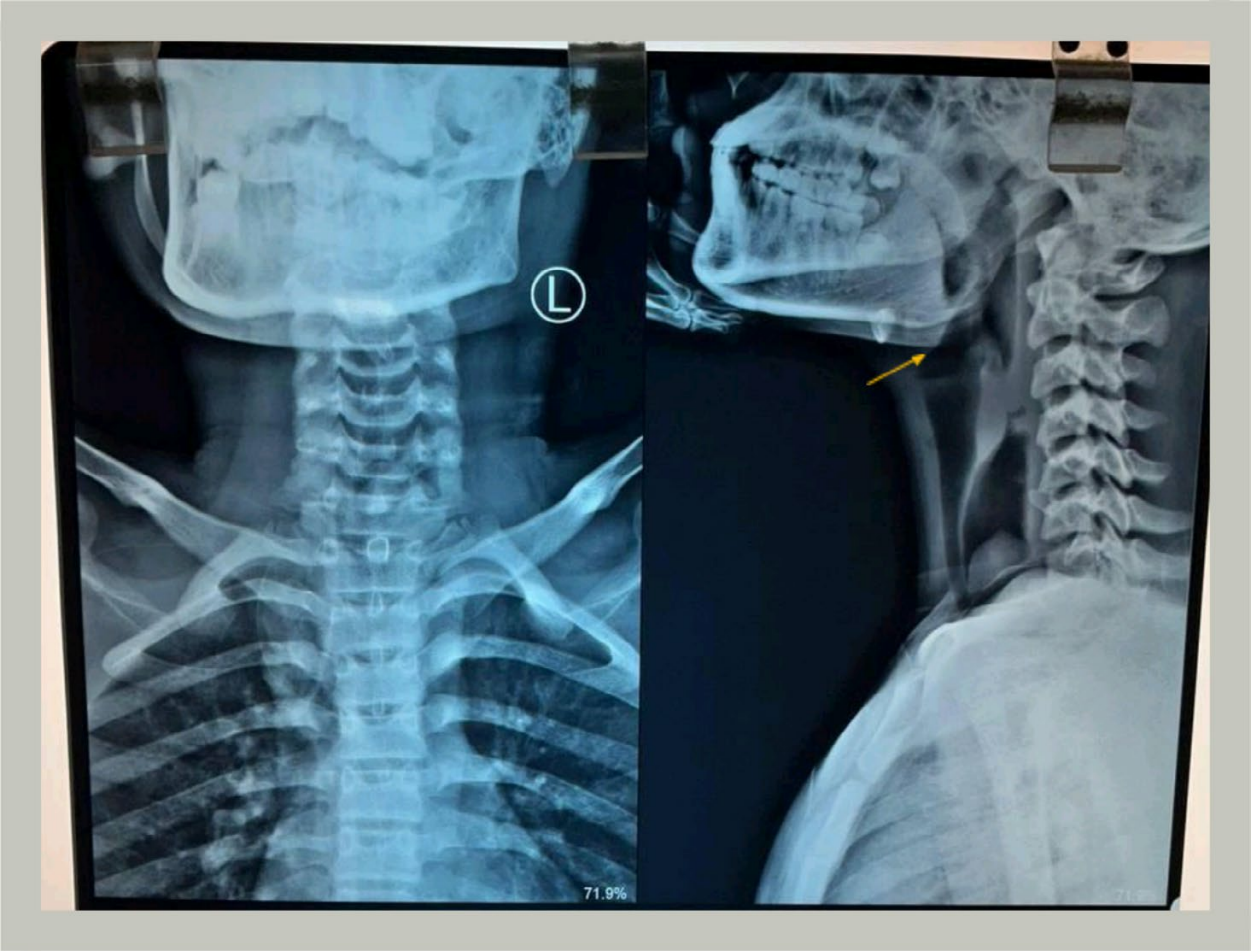
Anterioposterior (left) and lateral (right) radiographic views of head, neck, and upper chest.

**FIGURE 2:**
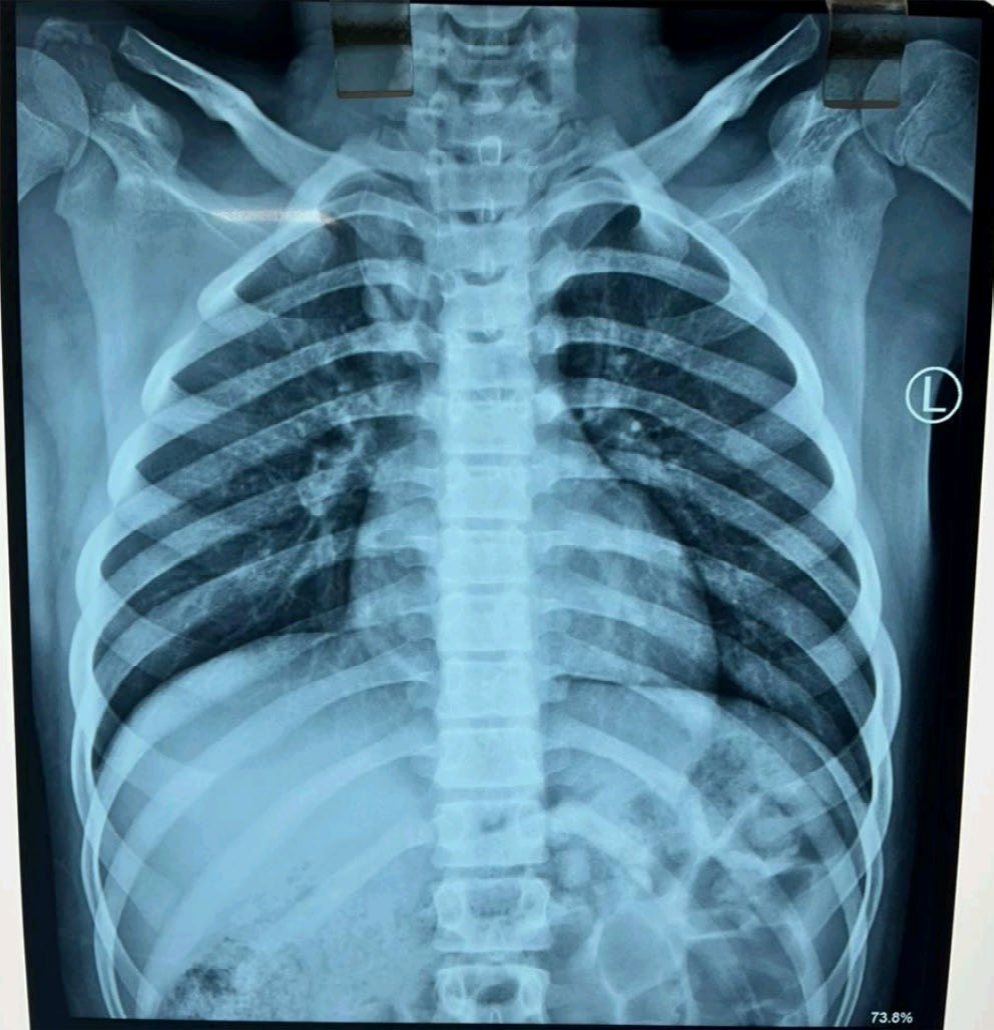
Chest X‐ray PA view.

A frontal radiograph and lateral radiograph (X‐ray) of the head and neck revealed a globular, radiopaque shadow at the C7‐T1 vertebral level consistent with a foreign body lodged in the esophagus (Figure 1). A Chest X‐ray was also done which revealed hyperdense mass at level of 7th cervical and 1St thoracic vertebrae (Figure 2). Chest looks normal.

## 5 Therapeutic Intervention

The patient was transferred to the operating theater where rigid esophagoscopy was performed under general anesthesia using a pediatric‐sized Nigus‐type esophagoscope, 25 cm. A whole lemon was visualized, firmly impacted in the distal esophagus. Adequate illumination and continuous visualization were maintained throughout the procedure to minimize mucosal trauma. Initial attempts were made to gently mobilize and extract the foreign body. However, due to firm impaction against the esophageal wall, careful dislodgment was performed before further manipulation. During the attempted extraction, the lemon dislodged and slipped distally into the stomach, thereby relieving the obstruction. Special precaution was taken to prevent mucosal injury, including gentle handling to avoid bleeding and laceration. Horizontally oriented foreign bodies were repositioned into vertically aligned to facilitate safer removal. The scope and patient positioning were adjusted as needed to optimize the angle of approach. Lubrication with xylocaine jelly was applied to the distal tip of the scope to reduce friction and minimize mucosal trauma. Following distal advancement into the stomach, the esophageal mucosa was carefully reinspected for evidence of injury. No mucosal tear, bleeding, or perforation was observed. In view of the absence of suspected injury, blind nasogastric tube insertion was also avoided. The patient was closely monitored postoperatively for any delayed complication.

## 6 Conclusions and Results (Outcome and Follow‐Up)

Postoperative recovery was uneventful. The patient was monitored in the intensive care unit (ICU), where his symptoms resolved promptly following successful endoscopic intervention. No signs of perforation, mucosal injury, or airway compromise were observed. He was kept nil per os (NPO) for a brief period, observed overnight, and discharged the following day in stable condition.

Caregiver received follow‐up counseling focused on dietary safety, monitoring behavioral cues, and strategies to prevent recurrence of future ingestion episodes. A plan for long‐term behavioral support and home safety reinforcement was discussed.

## 7 Discussion

This case underscores the diagnostic challenge posed by the atypical presentation of esophageal foreign body ingestion in nonverbal adolescents with intellectual disability. Despite initial symptoms suggestive of a seizure episode—such as abnormal movements, persistent drooling, and agitation—the absence of classical postictal confusion and lack of rhythmic convulsions prompted a re‐evaluation of the diagnosis. The patient's repeated attempts to insert his fingers into his mouth, resulting in superficial oral mucosal abrasions, along with self‐injurious behavior like head‐banging, served as important clinical clues indicating distress. It was the clinical intuition and experience of a senior emergency physician that led to consideration of a non‐neurological etiology. This case serves as a reminder that in fast‐paced emergency settings, where rapid diagnosis is often essential, clinicians must avoid cognitive biases like premature closure, especially in nonverbal or intellectually disabled individuals who may present atypically.

The patient in this report was a 17‐year‐old male. This age is slightly higher than the mean age (9.8 ± 3.5 years) reported in a study by Francesca Destro et al. among intellectually disabled and nonverbal (ID‐ND) patients, which also noted a male predominance (*p* = 0.02) [3]. This difference may be attributed to the extended developmental and behavioral vulnerability of adolescents with intellectual disabilities, who often retain risk profiles similar to much younger children. Furthermore, limited access to caregiver education, behavioral training, and rehabilitative services in resource‐limited settings such as Eastern Nepal likely contributes to the persistence of such risks into adolescence.

In this case, the impacted foreign body was a whole lemon—an unusual finding. A study by Suraj KC et al. reported coins as the most commonly ingested foreign objects in over 60% of pediatric cases, followed by toys (10%) and jewelry (7%) [4]. The rarity of this case may be attributed to vigilant supervision by the patient's family, who had taken preventive measures by keeping common small objects like coins, toys, and jewelry out of reach. However, unconventional objects like a lemon were not anticipated as potential risks.

Interestingly, the lemon was lodged at the C7–T1 vertebral level, differing from the typical site of impaction described by Krishna Chandra Rijal et al., who found the cricopharyngeal region (C5–C6 in adults) to be the most common site of esophageal foreign body impaction (78.26%) [2]. This deviation may be due to the unusually large size of the object and the patient's forceful swallowing attempts, pushing the object further down the esophagus.

The clinical presentation in this case also contrasted with that reported by Brian M. Fung et al., where the most common symptoms of esophageal foreign body ingestion were chest pain, dysphagia, foreign body sensation, odynophagia, sialorrhea, and the urge to spit up secretions [5]. The lack of these classic symptoms in our patient can be explained by his intellectual disability and nonverbal status, which significantly impaired his ability to communicate both the event and subsequent discomfort, leading to delayed diagnosis.

The patient underwent rigid esophagoscopy under general anesthesia within 24 h of presentation. This aligns with findings by Brian M. Fung et al., who emphasized that endoscopic intervention within 24 h is critical, as delays increase the risk of complications [5]. However, this contrasts with a study by Salvatore Olivia et al., which noted that only 10%–20% of such cases require endoscopic removal [6]. The difference in our case is likely due to the size and shape of the ingested object—a whole lemon—necessitating urgent intervention.

Post‐operatively, the patient had an uneventful recovery and was discharged on the third day, consistent with the findings of Omer Topaloglu et al., who reported a mean hospital stay of 3.3 days for symptomatic patients [7]. The timely diagnosis and intervention in this case likely contributed to the absence of complications and reduced hospital stay.

A similar case was described by Topaloglu et al., who reported esophageal impaction of two large spherical foreign bodies in an intellectually disabled adolescent, including a walnut and an identification wristband. Despite attempts at rigid esophagoscopic extraction, retrieval and distal displacement into the stomach were unsuccessful due to the size and impaction of the object, ultimately requiring surgical removal via cervical esophagotomy. In contrast, our case involved a single large foreign body (whole lemon) that initially resisted extraction but was successfully displaced into the stomach during rigid esophagoscopy, relieving the obstruction without mucosal injury. This comparison highlights the variability in management strategies depending on the size, consistency, number, and degree of impaction of the foreign body, while endoscopic intervention remains the first‐line treatment; surgical management remains an essential alternative when endoscopic retrieval or safe distal advancement is not feasible. Additionally, the report by Topoglou et al. underscores the importance of actively addressing the presence of multiple foreign bodies, as additional ingested objects may remain undetected after the removal of the initially identified object. Therefore, thorough endoscopic evaluation and careful post‐operative surveillance are crucial to avoid missed foreign bodies and prevent reoccurrence complications. Furthermore, Topaloglu et al. emphasize the importance of post‐operative caregiver education and behavioral supervision, which was also addressed in our patient to reduce the risk of recurrence.

## Author Contributions

**Riya Thapa:** conceptualization, data curation, formal analysis, funding acquisition, investigation, methodology, project administration, resources, software, supervision, validation, visualization, writing – original draft, writing – review and editing. **Utsav Sitaula:** conceptualization, data curation, formal analysis, funding acquisition, investigation, methodology, project administration, resources, software, supervision, validation, visualization, writing – original draft, writing – review and editing. **Raju Basnet:** conceptualization, data curation, formal analysis, funding acquisition, investigation, methodology, project administration, resources, software, supervision, validation, visualization, writing – original draft, writing – review and editing. **Ajay Kumar Yadav:** conceptualization, investigation, methodology, project administration, resources, supervision, validation, visualization. **Gyanendra Bahadur Malla:** conceptualization, methodology, resources, supervision, validation, writing – review and editing.

## Funding

The authors have nothing to report.

## Disclosure

Patient's Perspective: The patient and his family reported significant relief and satisfaction following the endoscopic intervention. Due to communication limitations, the patient's perspective was primary obtained through caregiver reporting. They expressed gratitude for the diagnosis and treatment.

## Consent

Written informed consent was obtained from the patient for publication of this case. report and any accompanying images, in accordance with the SCARE guidelines. It will be available whenever requested by the Editor in Chief.

## Conflicts of Interest

The authors declare no conflicts of interest.

## Data Availability

The data that support the findings of this study are available on request from the corresponding author. The data are not publicly available due to privacy or ethical restrictions.
